# Medical Application of Hydrogen in Hematological Diseases

**DOI:** 10.1155/2019/3917393

**Published:** 2019-11-28

**Authors:** Liren Qian, Zhengcheng Wu, Jian Cen, Sergiu Pasca, Ciprian Tomuleasa

**Affiliations:** ^1^Department of Hematology, The Sixth Medical Center, Chinese PLA General Hospital, China; ^2^Department of Medical Service, The Sixth Medical Center, Chinese PLA General Hospital, China; ^3^Department of Hematology, Ion Chiricuta Oncology Institute, Cluj-Napoca, Romania; ^4^Iuliu Hatieganu University of Medicine and Pharmacy, Cluj-Napoca, Romania

## Abstract

Hydrogen gas has been reported to have medical efficacy since the 1880s. Still, medical researchers did not pay much attention to hydrogen gas until the 20th century. Recent research, both basic and clinical, has proven that hydrogen is an important physiological regulatory factor with antioxidative, anti-inflammatory, and antiapoptotic effects. In the past two decades, more than 1000 papers have been published on the topic, including organ ischemia-reperfusion injury, radiation injury, diabetes, atherosclerosis, hypertension, or cancer. We have previously hypothesized and proven the therapeutic effects of hydrogen gas in graft-versus-host disease following stem cell transplantation. In the current manuscript, we present the clinical advances of hydrogen gas in hematological disorders.

## 1. Introduction

Hydrogen gas is the simplest molecule in nature which is formed by two hydrogen atoms. Previously, hydrogen gas is thought to be chemically inactive and cannot react with other substances easily at room temperature which easily make medical scientists thought it might be a kind of physically inert gas. There were only three papers published have demonstrated that hydrogen gas is a kind of therapeutic gas in the last two centuries.

The first paper was reported in 1888 by Pilcher [1]. They used hydrogen gas to locate visceral injuries by insufflating the gastrointestinal canal avoiding unwarranted surgeries. However, the authors used the physical character of hydrogen gas without finding out its biomedical ability. The first paper reported the biomedical ability of hydrogen gas was published in 1975 by Dole et al. [2]. In their paper, they make the mice with skin squamous cell carcinoma breathe a mixture (2.5% O_2_ and 97.5% H_2_) at a total of 8.28 atmospheres for 2 weeks. Amazingly, the volume of the skin squamous cell carcinoma reduced a lot with no deleterious consequences. They explained that the mechanism behind the phenomenon may be that H_2_ is a free radical decay catalyzer for the first time. In the seminal paper, they hypothesized for the first time that hydrogen might act to scavenge the ^·^OH radical, also demonstrated by Buxton et al. until 1988 in cell free systems [3]. In 2001, Gharib et al. further reported the antioxidative effect of hydrogen gas in vivo [4]. They reported that inhalation of 0.7 MPa H_2_ has therapeutic effects on schistosomiasis-associated chronic liver inflammation and thought the anti-inflammatory effects of hydrogen may be due to its scavenging hydroxyl radical ability.

However, these papers do not attract too much attention until 2007, when Ohsawa et al. demonstrated the selective antioxidative ability and therapeutic effects of hydrogen gas on cerebral ischemia [5]. This article has had great influence in the area of hydrogen medicine. The number of articles in hydrogen biology afterwards increased sharply. Since 2007, hydrogen gas was used in 300 kinds of diseases. Most of them are oxidative stress-related or inflammatory diseases, including tissue/organ ischemia-reperfusion, radiation-induced injury, and toxicity of chemotherapy [6]. Despite many inaccuracies, selective free radical scavenging ability is still the widely accepted mechanism. In addition, its antiapoptotic and anti-inflammatory effects also attracted scientific researchers' attention in recent years. Herein, we focus on offering an overview of major scientific and clinical advances of hydrogen in hematological diseases.

## 2. Graft-versus-Host Disease (GVHD)

Graft-versus-host disease (GVHD) is one of the main complications of hematopoietic stem cell transplantation (HSCT) which was traditionally divided into acute and chronic GVHD based on the timing of the onset of GVHD symptoms [7]. However, this dividation is not precise because some acute GVHD manifestations occur after 100 days posttransplantation and some patients may manifest classic signs of chronic GVHD before day 100 [7]. Therefore, the Center for International Bone Marrow Transplant Registry (IBMTR) criteria updated the classification of GVHD [8–10]. There are three stages in the current mechanism of acute GVHD, in which inflammatory cytokines released play critical roles. Cytokines like tumor necrosis factor (TNF)-*α*, interleukin- (IL-) 1, and IL-6 may directly attack various host tissues which lead to the clinical manifestations of acute GVHD. Harmful free radicals can also be produced by activated cells which may injure cells and tissues [11]. It has been proven that hydrogen has antioxidative and anti-inflammatory effects [6]. Therefore, we hypothesized that hydrogen may exert therapeutic effects on acute GVHD [12]. We then tested and proved the hypothesis in an acute GVHD mouse model [13]. In our study, clinical syndromes of acute GVHD were significantly improved by hydrogen. The recovery of white blood cells was also promoted by hydrogen treatment. Cytokines like TNF-*α* and IL-2 which play critical roles in the development of acute GVHD were also regulated by hydrogen. Following our research, Yuan et al. also proved hydrogen's therapeutic effects on acute GVHD and got similar results in the experiments [14].

As the other type of GVHD, chronic GVHD has become the leading cause of nontransplantation-related death in recent years due to its rising incidence rate [15, 16]. The exact mechanism of chronic GVHD is still unclear. Inflammatory factor imbalance and fibrosis formation were widely accepted as the main mechanism of chronic GVHD [17]. Due to the anti-inflammatory and antifibrosis effects of hydrogen, we hypothesized that it may have therapeutic effects on chronic GVHD. In our study, survival rate of chronic GVHD mice was increased and the skin lesions were reduced by hydrogen [18]. Besides, we also published a case report of a patient with severe chronic graft-versus-host disease successfully treated by drinking hydrogen-rich water which makes hydrogen as a promising method for treating GVHD [19]. A 54-year-old Chinese man with myelodysplastic syndromes developed chronic GVHD 3 years after allo-HSCT that was refractory to prednisone and tacrolimus. After drinking hydrogen-rich water, prednisone and tacrolimus were tapered in three months and the symptoms were relieved. Also, we have carried out a clinical trial to further investigate the efficacy and safety of hydrogen on chronic GVHD (ClinicalTrials.gov number, NCT02918188).

## 3. Hemorrhage

The incidence of hemorrhage in our daily life is high. Severe hemorrhage is a major cause of death in trauma and accident. Lethal hemorrhage is also the leading cause of death in war injury [20, 21]. During uncontrolled hemorrhage, hemostasis and reinfusion by blood transfusion are always the most important. Still, in many scenarios like severe trauma and far away from hospitals, stopping bleeding completely is impossible, thus affecting hemodynamic stability. Free radicals and inflammatory cytokines generated during the process of hypoperfusion, organ ischemia, and hypoxia may exacerbate organ injuries. Therefore, several researchers have studied the protective role of hydrogen in hemorrhage. Kohama et al. have proved the potential therapeutic effects of inhaling 1.3% hydrogen in acute lung injury induced by hemorrhagic shock [22]. They reported the modulation role of hydrogen in the messenger RNAs for IL-1*β* and TNF-*α* and decreased activation of NF-*κ*B. It was suggested that hydrogen may exert its therapeutic effects by NF-*κ*B-mediated pathways. Du et al. also reported that hydrogen may ameliorate lung injury induced by uncontrolled hemorrhagic shock in rats via its anti-inflammation and antioxidative abilities [23]. The same group also demonstrated the same protective role of hydrogen in rat's intestinal injury in uncontrolled hemorrhagic shock [24]. In the brain hemorrhage rat models, Zhan et al. and Hong et al. also showed that hydrogen may ameliorate the brain injury caused by subarachnoid hemorrhage [25, 26]. In another paper by Matsuoka et al., they found that hydrogen gas inhalation may not only prevent vital organ damage caused by irreversible hemorrhagic shock but also stabilize hemodynamics increasing the survival rate in posthemorrhagic shock [27]. The survival rate in the hydrogen group was significantly higher than the control group at 6 hours after resuscitation (80% vs. 30%). Blood pressure was stabilized, and metabolic acidosis was well rectified in the hydrogen group at 2 hours after resuscitation. Thus, it was demonstrated that hydrogen has potential therapeutic effects in different organs' hemorrhage. But its mechanism is still not quite clear, mostly based on its anti-inflammation and antioxidative abilities. Whether hydrogen can activate signal pathways directly still needs assessment by rigorous experimental data.

## 4. Aplastic Anemia

Aplastic anemia is a kind of disease with bone marrow failure disorder which has high lethality rate [28]. Most patients die from severe infection and hemorrhage. Radiation, pathogenic microorganism, and chemical agents are nevertheless linked to the etiopathogenesis of aplastic anemia [29, 30]. Even if, most cases are classified as idiopathic [29]. Autoimmune response caused by activated T cells and hypersecretion of inflammatory cytokines like IFN-*γ* and TNF-*α* occupy the dominant position in the development of aplastic anemia [28]. The autoimmune response results in the destruction of hematopoietic stem and progenitor cells [30]. Abnormalities of the levels of TNF-*α* and IL-6 have been proven playing important roles in the pathogenesis of aplastic anemia [31–33]. Oxidative injury is also linked to the development of aplastic anemia [34, 35]. These studies make us to hypothesize that hydrogen may be effective on aplastic anemia [36]. Zhao et al. tested this very hypothesis and assessed the efficacy of hydrogen in aplastic anemia in a mice model [37]. They showed that hydrogen may improve the body weight, number of peripheral blood cells, and the bone marrow microenvironment by decreasing the levels of TNF-*α*, IFN-*γ*, and IL-6. Interestingly, the ratio of CD4/CD8 increased to normal gradually. This indicates that hydrogen may be a therapeutic agent for aplastic anemia by regulating its inflammation balance.

## 5. Platelet Aggregation

Platelet is one of the most important factors in thrombogenesis. Sometimes, thrombus is protective when the adult organisms were wounded. But in some circumstances, it may cause severe thrombotic diseases including cerebral infarction or pulmonary embolism. Platelet aggregation is thought to be closely linked to ^·^OH [38]. Takeuchi et al. have hypothesized that hydrogen may inhibit collagen-induced platelet aggregation via the reduction of ^·^OH [39]. They found that hydrogen-rich saline may inhibit collagen-induced platelet aggregation in healthy volunteers' blood samples. The efficacy of hydrogen was also demonstrated by the same group in murine model of the disease [39]. Recently, Wang et al. have reported that hydrogen may inhibit platelet activation and prevent thrombosis formation [40]. In their study, they found that hydrogen-saturated water (concentration of 0.6 mM) inhibited ADP-induced platelet aggregation at doses ranging from 10 *μ*l to 50 *μ*l and collagen-induced rat platelet aggregation at doses ranging from 20 *μ*l to 50 *μ*l. Thrombus dry weights were less in the hydrogen group than in the control group in FeCl_3_-induced arterial thrombosis models. Occlusion time was also prolonged by hydrogen. The mechanism is based on the antioxidant property via the subsequent NO/cGMP/PKG/ERK pathway. These studies showed that hydrogen can be used in the scenarios where platelets are abnormally activated, as is the case for thrombotic diseases.

## 6. Lymphoma

An important external factor in the pathogenesis of lymphomas is ionizing radiation (IR), which can initiate and promote tumor progression [41–43]. Free radicals, especially ^·^OH, play critical roles in both radiation-induced injuries and the pathophysiology of tumors. Zhao et al. showed that radiation-induced thymic lymphoma in BALB/c mice may be reduced by hydrogen [44]. They found that hydrogen may decrease the radiation-induced thymic lymphoma rate and significantly delayed the development of lymphoma after the split dose irradiation. This research thus elucidates whether hydrogen may also exert therapeutic effects on hematopoietic malignancies, similar to the first report regarding its effects on skin squamous cell carcinoma in the 1970s [2]. In the current manuscript, we hypothesized that the mechanism may be the radioprotective when assessing the effects of hydrogen by its antioxidative ability. However, whether hydrogen can suppress lymphoma directly, as reported in the 1975 manuscript, is still not clear and should be studied further [2]. If hydrogen could also exert therapeutic effects on lymphoma directly, it may have effects on other types of cancers.

## 7. Conclusion

**Figure 1:**
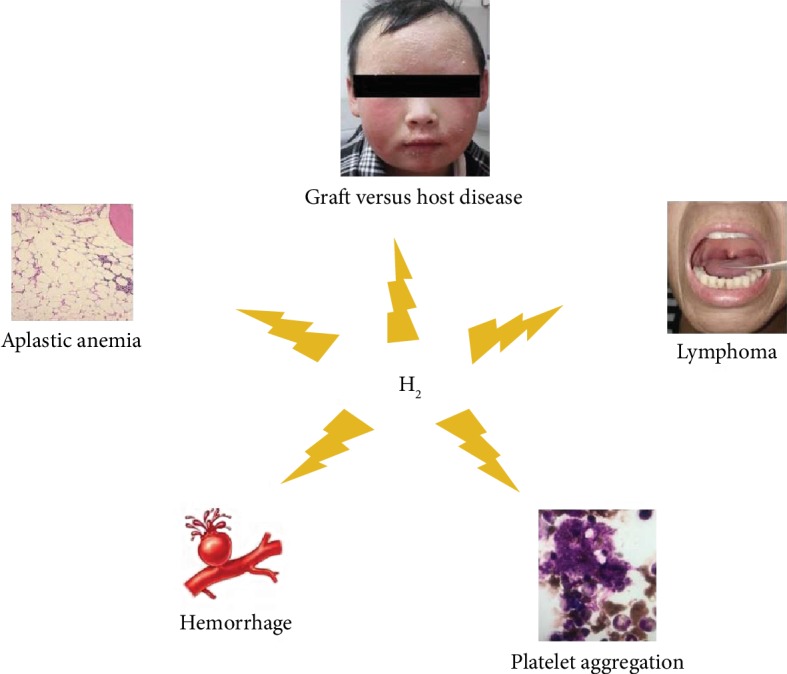
Medical application of hydrogen in different hematological diseases.

With better understanding of the mechanism of hematological diseases and development of targeted therapeutic drugs, an increasing number of novel therapeutics have been developed. However, there are still many unsolved problems which need more research. Hydrogen gas may be a frontline therapeutic, but only a few researchers have focused in this direction. Although it has been demonstrated in many diseases in basic researches and clinical researches, it has been applied to only a few hematological diseases (Figure 1). There are still many unexplored areas of hematological researches. Many hematological disorders are linked to oxidative injury and inflammation, as is the case for hemolytic diseases, thrombocytopenia, and hematopoietic malignancies; whether hydrogen may exert therapeutic effects on these diseases is still uncertain.

There are some advantages of hydrogen in hematological diseases. Firstly, hydrogen has few side effects. The mixture of hydrogen and helium has been used in diving for a long time, which showed its safety [45]. Secondly, the molecules of hydrogen are very small and have a strong penetrating ability, which can quickly penetrate the biofilm and reach a high concentration within the cell for therapeutic effects [5]. Thirdly, the price of hydrogen is very low and is easy to be obtained, while some drugs treating hematological diseases are very expensive. However, hydrogen is explosive and the concentration of hydrogen in water is unstable which are the disadvantages. Hematological diseases always processed quickly, but the therapeutic effects of low concentration hydrogen is always mild and high concentration of hydrogen is explosive. These are all problems that need us to figure out.

There are still some enigmas of hydrogen effects. For example, hydrogen gas can be produced in rodents and human intestines. A maximum of 12 liters of hydrogen are able to be made in human intestines [46, 47]. The amount of hydrogen produced by intestinal bacteria is much more than that taken by water or gas, but the exogenously administered hydrogen demonstrates a prominent effect. The concentration of hydrogen reached maximum within 15 mins after oral administration of hydrogen water and then returned to base levels in 30 mins [48]. Whether exogenous hydrogen may exert its effects as a signal molecule or whether oral hydrogen-rich water may exert effects by regulating intestinal microecology is uncertain. Other mechanisms of hydrogen action still need further studies, with H_2_ being a new medical gas which may pose a great potential. H_2_ as the new medical gas has not attracted much attention from hematologists. We hope that more patients with hematological diseases can benefit from this ancient medical gas.
